# Safety and immunogenicity of an HIV-1 gp120-CD4 chimeric subunit vaccine in a phase 1a randomized controlled trial

**DOI:** 10.1016/j.vaccine.2021.05.090

**Published:** 2021-06-29

**Authors:** Joel V. Chua, Charles Davis, Jennifer S. Husson, Amy Nelson, Ilia Prado, Robin Flinko, Ka Wing J. Lam, Lydiah Mutumbi, Bryan T. Mayer, Dan Dong, William Fulp, Celia Mahoney, Monica Gerber, Raphael Gottardo, Bruce L. Gilliam, Kelli Greene, Hongmei Gao, Nicole Yates, Guido Ferrari, Georgia Tomaras, David Montefiori, Jennifer A. Schwartz, Timothy Fouts, Anthony L. DeVico, George K. Lewis, Robert C. Gallo, Mohammad M. Sajadi

**Affiliations:** a Division of Clinical Care and Research, Institute of Human Virology, University of Maryland School of Medicine, Baltimore, MD, USA; b Division of Vaccine Research, Institute of Human Virology, University of Maryland School of Medicine, Baltimore, MD, USA; c Vaccine and Infectious Disease Division, Fred Hutchinson Cancer Research Center, Seattle, WA, USA; d Public Health Sciences Division, Fred Hutchinson Cancer Research Center, Seattle, WA, USA; e Duke Human Vaccine Institute, Duke University School of Medicine, Durham, NC, USA; f Intralytix, Columbia, MD, USA; g Advanced BioScience Laboratories, Rockville, MD, USA; h Global Virus Network, Baltimore, MD, USA; i Division of Basic Science, Institute of Human Virology, University of Maryland School of Medicine, Baltimore, MD, USA

**Keywords:** HIV, Vaccine, Chimeric subunit vaccine, Full-length single chain (FLSC), CD4i

## Abstract

A major challenge for HIV vaccine development is to raise anti-envelope antibodies capable of recognizing and neutralizing diverse strains of HIV-1. Accordingly, a full length single chain (FLSC) of gp120-CD4 chimeric vaccine construct was designed to present a highly conserved CD4-induced (CD4i) HIV-1 envelope structure that elicits cross-reactive anti-envelope humoral responses and protective immunity in animal models of HIV infection. IHV01 is the FLSC formulated in aluminum phosphate adjuvant. We enrolled 65 healthy adult volunteers in this first-in-human phase 1a randomized, double-blind, placebo-controlled study with three dose-escalating cohorts (75 µg, 150 µg, and 300 µg doses). Intramuscular injections were given on weeks 0, 4, 8, and 24. Participants were followed for an additional 24 weeks after the last immunization. The overall incidence of adverse events (AEs) was not significantly different between vaccinees and controls. The majority (89%) of vaccine-related AE were mild. The most common vaccine-related adverse event was injection site pain. There were no vaccine-related serious AE, discontinuation due to AE, intercurrent HIV infection, or significant decreases in CD4 count. By the final vaccination, all vaccine recipients developed antibodies against IHV01 and demonstrated anti-CD4i epitope antibodies. The elicited antibodies reacted with CD4 non-liganded Env antigens from diverse HIV-1 strains. Antibody-dependent cell-mediated cytotoxicity against heterologous infected cells or gp120 bound to CD4+ cells was evident in all cohorts as were anti-gp120 *T*-cell responses. IHV01 vaccine was safe, well tolerated, and immunogenic at all doses tested. The vaccine raised broadly reactive humoral responses against conserved CD4i epitopes on gp120 that mediates antiviral functions.

## 1 Introduction

Despite more than three decades of research, a highly effective preventative vaccine against the human immunodeficiency virus 1 (HIV-1) is still not available. A vaccine that elicits antibody responses to the viral envelope spike is expected to be protective. Such responses could prevent or suppress infection by direct neutralization or Fc-mediated effector functions such as antibody-dependent cell-mediated cytotoxicity (ADCC), phagocytosis, or trogocytosis [1], [2], [3]. However, a major challenge to this concept stems from the capacity of HIV to evolve mutational escape from humoral immunity. Antigenic domains on the surfaces of free virions readily acquire such changes in the face of immune pressure.

Potential opportunities to overcome this hurdle are presented by the nature of HIV attachment and entry. HIV virions express surface heterotrimers comprised of two components, gp120 and gp41. During attachment, the gp120 component of the envelope spike forms a transition state structure upon virion binding to the host cell CD4 receptor. This structure is distinguished by the presentation of extremely conserved, CD4-induced (CD4i) epitopes, some of which perform the critical role of binding to cell coreceptors (primarily CCR5) that trigger membrane fusion and viral entry [4], [5]. CD4i epitopes can be immunoreactive in multiple scenarios during spreading infection. For example, allosteric mechanisms propagate the expression of CD4i epitopes across virion surfaces after host cell attachment occurs [6], [7]. Further, CD4i epitopes are expressed at the contact interfaces of fusing infected and uninfected cells and across the surfaces post-fusion cell pairs [8], [9], [10]. Consequently, antibodies recognizing CD4i epitopes have opportunities to be broadly antiviral if present before exposure, holding potential utility for HIV vaccine development.

In accordance with this concept, anti-CD4i antibodies are known to mediate neutralizing activity as well as various Fc-mediated effector functions including ADCC, phagocytosis and trogocytosis [10], [11], [12], [13], [14], [15], [16], [17]. The structural basis for the translation of anti-CD4i antibody binding into antiviral activity has been studied extensively [11], [13], [14], [18], [19], [20]. CD4i epitopes are naturally immunogenic, frequently eliciting antibody titers in HIV-infected persons [21], [22], [23], [24], [25], [26]. Anti-CD4i antibody responses fortuitously raised by HIV envelope-based vaccines in human trials were linked with reduced risk of infection [27], [28], [29]. In addition, similar responses correlated with protection or control of viremia in HIV envelope-vaccinated macaques challenged with simian immunodeficiency viruses (SIV) or chimeric SIV expressing the HIV envelope (SHIV) [30], [31].

A full-length single chain (FLSC) of gp120-CD4 chimera subunit vaccine was developed to exploit the potential vulnerabilities of transition state/CD4i envelope structures. FLSC is a subunit vaccine encoded by a synthetic gene expressing a human codon-optimized, full-length HIV (BaL isolate) gp120 sequence joined at its C terminus to the N terminus of domains 1 and 2 of human CD4 (CD4D1D2) via a flexible 20 amino acid linker that covalently links the gp120 and CD4 portions [32]. The gp120 sequences are translated as the N terminus of the chimera and the CD4 sequence at the C terminus. This construction allows the gp120 and CD4 moieties to form a stable intra-chain binding interaction replicating the gp120 transition state structure [32]. The detected antigenic and biochemical characteristics of FLSC are consistent with structural information from crystallographic and cryoelectron microscopic studies of gp120-CD4 complexes [32], [33], [34], [35]. However, soluble CD4 elicits a greater array of CD4i epitopes in FLSC versus intact envelope trimers [18]. Proof-of-concept studies performed in rhesus macaques repeatedly demonstrate that chimeric gp120-rhesus CD4 complexes (rhFLSC) in various adjuvants and prime boost protocols induce antibodies to CD4i epitopes, leading to post-infection control of viremia for high-dose challenges [31], [36] and delayed acquisition for repeat low-dose challenges [30], [37]. Protection in rhFLSC-vaccinated animals did not track with the cross-reactive serum neutralizing component of humoral responses but did correlate with ADCC activity. This latter relationship is in accordance with other nonhuman primate studies of HIV vaccine concepts linking protection with Fc-mediated effector functions (see for examples [30], [38], [39], [40], [41], [42], [43], [44], [45], [46]. FLSC-vaccinated animals did not exhibit autoreactive anti-CD4 autoantibodies or other detectable alterations in circulating CD4 + T cells [47]. A recent study showed that rhFLSC could be used as a component of a vaccination regimen that afforded protection in macaques via trained immunity [48]. IHV01 is the FLSC vaccine (with human CD4D1D2) formulated in aluminum phosphate adjuvant (Alum).

In the current study, the safety, tolerability, and immunogenicity of IHV01 was assessed in a human, randomized, double-blind, placebo-controlled dose-escalating trial (ClinicalTrials.gov NCT02756208).

## 2 Methods

### 2.1 Study design and participants

This phase 1a, dose-escalating, randomized, double-blind, placebo-controlled trial on the safety, tolerability, and immunogenicity of IHV01 in adult volunteers was conducted at a single site, Institute of Human Virology, Baltimore, Maryland, USA. The primary objectives were to evaluate safety and tolerability of intramuscular (IM) administration of the IHV01 at three different doses (75 µg, 150 µg, and 300 µg). This trial was divided into three cohorts with enrollment done sequentially from lowest to highest dosing [Table 1].

**Table 1:** Study design with immunization schedule and volunteer allocation.

**Cohort**	**Route of Administration**	**N** **Vaccine/Control**	**Vaccine Dose**	**Vaccination Schedule in Weeks (Days)**			
				**0**	**4 (28)**	**8 (56)**	**24 (168)**
1	IM	15	0.25 ml (75 µg)	IHV01	IHV01	IHV01	IHV01
		5	0.25 ml	Saline	Saline	Saline	Saline
2	IM	15	0.5 ml (150 µg)	IHV01	IHV01	IHV01	IHV01
		5	0.5 ml	Saline	Saline	Saline	Saline
3	IM	15	1.0 ml (300 µg)	IHV01	IHV01	IHV01	IHV01
		5	1.0 ml	Saline	Saline	Saline	Saline
**TOTAL**		**60**					

Eligible participants were HIV-1 uninfected healthy volunteers 18–45 years of age with low risk of acquiring HIV infection and had CD4 count within the normal range. Key exclusion criteria included pregnancy, breastfeeding women, presence of HIV (antibody), hepatitis B (surface antigen), hepatitis C (PCR), and prior receipt of HIV vaccine. Participants were eligible if their CD4 count was within the normal range and CD4 percentage within 20% of the normal range of the clinical laboratory values. All participants provided written informed consent. The University of Maryland Institutional Review Board approved this study.

### 2.2 Vaccine

IHV01 is comprised of the FLSC gp120-CD4 chimera subunit antigen formulated in aluminum phosphate (AlPO_4_) adjuvant. The drug product consists of 0.3 mg/mL of purified cGMP drug substance formulated with AlPO_4_ at 2.4 mg/mL in binding buffer (mannitol 40 mg/mL; sodium acetate 5 mM, pH 6.2). The vaccine antigen is purified from clarified harvest fluids of a 200L bioreactor culture of a FLSC protein producing G293H cell line, a derivative of the HEK 293 human embryonic kidney cell line. This vaccine product was manufactured by the Institute of Human Virology (IHV) in partnership with Profectus BioSciences, Inc. (since acquired by another company) for this study. IHV01 Lot # 14MM-022 was used for this study and was stored between 2 °C an 8 °C in the IHV research pharmacy. Placebo consisted of saline.

### 2.3 Randomization and blinding

In each dose-escalating cohort, blocks of four eligible participants were assigned in a 3:1 ratio to either intervention or placebo control groups using block randomization design. Participants and study staff, except study pharmacist preparing the injections, were blinded to treatment allocation within each cohort.

### 2.4 Procedures

Eligible participants received intramuscular injections with IHV01 (intervention group) or placebo (control group) on weeks 0, 4, 8, and 24. Injections were administered intramuscularly in the deltoid muscle of the participant’s non-dominant arm (unless preferred otherwise) using a 22 gauge 25 mm (1 in.) needle. Participants returned for a study visit 2 weeks post-vaccination and were all followed for an additional 24 weeks after the last immunization. Blood samples were collected for safety labs and immunogenicity assays on days of vaccination, 2 weeks after each vaccination, and on weeks 28, 36, 42, and 48.

Participants recorded local and systemic reactions on a diary card for 7 days after vaccination. Adverse events (AEs), including serious AEs (SAEs) occurring throughout the trial were recorded. Safety laboratory assessments included complete blood count with differential, serum electrolytes, liver, and renal function tests, and CD4 + T-cell counts were obtained at each study visit from screening through week 48. Women of childbearing potential had serum pregnancy tests done at screening, and urine pregnancy tests were performed prior to each immunization and at study completion. HIV infection status was determined at screening and at week 48 using commercially available HIV-1 RNA PCR assay and 4th generation HIV-1/2 antibody/antigen with cascade reflex to supplementary differentiation test.

An independent safety review board assessed safety data beginning with the enrollment of the first volunteer into the study. The safety board reviewed blinded data and assessed the study prior to advancement to the next higher dose cohort and at regular scheduled intervals. Safety assessment included monitoring for unexplained CD4 cell count decline (confirmed by assays at least 4 weeks apart) of greater than 30% and corroborated by similar CD4% decline (30%). If five or more volunteers in any group had this unexplained CD4 decline, further immunization was to be halted and a safety board review was to be triggered.

### 2.5 CD4 *T*-cell analysis

CD4 count was monitored from baseline and at each study visit through study completion. Baseline CD4 and CD4% levels were calculated by taking the average of the screening and first vaccination visits. Fold-change average CD4 count (pre vs post) and difference in CD4% (pre vs post) were applied to determine whether there was an overall difference, but also calculated at specific time points to evaluate changes between baseline and subsequent post-vaccination time points. For each CD4 outcome, the standard deviation was estimated from a linear mixed effect model which considered the within-subject variation as a random effect. CD4 count fold-change was log-transformed in the model. Estimated standard deviations from these models have been previously established to determine unusual declines in CD4 levels [49]. To model changes in CD4 outcomes (CD4 percentage and log-transformed CD4 count) using predictors, linear mixed models were used with a random intercept to control for within-subject variation. Adjusted models were fit using vaccination group, time, and the interaction between group and time, as well as age, gender, and race. Model-estimated means and pairwise group tests—testing for differences in CD4 changes between vaccinated participants and participants receiving placebos—were calculated using the models which adjusted for demographic variables. P-values calculated from model pairwise group tests were adjusted for multiple testing using the Tukey adjustment method [50].

### 2.6 Immunogenicity assessments

The secondary objective of this trial was to evaluate vaccine immunogenicity according to the following measures: 1) anti-FLSC antibody responses (percent responders and response magnitudes); 2) cross-reactive anti-Env antibody responses (percent responders and response magnitudes in a panel of heterologous, CD4-nonliganded gp120, gp140 and gp70-scaffolded V1V2 antigens); 3) neutralizing antibody titers against a panel of heterologous viruses; 4) antibody-dependent cell-mediated cytotoxicity (ADCC) activity; 5) competitive antibodies (titers and percent responders) to CD4i epitopes on gp120 to better define the epitopes targeted by the antibody response.

Serum HIV-1 IgG titers against FLSC were measured in a Bio-Plex instrument (Bio-Rad) using a standardized custom HIV-1 Luminex assay [51], [27], [28]. Antibody titers were measured as median fluorescence intensity (MFI) from two wells and then averaged. Background adjustment was applied using MFI measured from wells with beads that include buffer instead of sample. Additionally, blank beads were included to estimate non-specific antibody binding. Net MFI was used as the binding antibody response magnitudes: the background-adjusted MFI minus background-adjusted blank (blank MFI). A positive response was determined using the following three criteria: (i) net MFI was greater than or equal to an antigen-specific control cutoff,(ii) net MFI was greater than 3 times the baseline (pre-vaccination) net MFI; and (iii) background-adjusted MFI was greater than 3 times the baseline background-adjusted MFI. Results were obtained for a single dilution of 1:50 for all antigens.

The HIV-1 Luminex assay described above was used to further assess the magnitude and breadth of responses against AIDS Reagent Program panels of CD4-nonliganded gp120, gp140, and gp70-scaffolded V1V2 antigens [Supplementary Table 1] comprising 32 Envs from five clades of HIV-1 [52]. Individual-specific and group-averaged magnitude-breadth (MB) curves [53] were used to display the breadth of binding antibody activity in terms of the percentage of antigens with log_10_ net MFI *<* x for the range of net MFI values, x**.** The area under the MB curve (AUC-MB score) was then used to summarize the magnitude and breadth for each individual at a given time point across a set of antigens. MFI values were set at 1 for antigens that did not elicit a response so only antigens showing a response contributed to the MB curves and AUC-MB.

Competition ELISAs were performed as described previously [21] to determine whether plasma samples contain antibodies against CD4i epitopes (A32, 17b, and N12i2) on FLSC. The monoclonal antibody (mAb) A32 targets Cluster A, the gp120 face occluded by gp41 in trimeric Env, while mAbs 17b and N12i2 target Cluster C, the co-receptor binding site. Briefly, 96-well plates pre-coated with FLSC (1 µg/ml) were incubated with the indicated concentrations of plasma samples premixed with a biotinylated anti-CD4i epitope mAb of half-maximum binding concentration for 30 min. Bound mAbs were then detected with HRP conjugated poly streptavidin (1:1000) and then the HRP substrate TMB [3, 3′,5 ,5′- tetramethyl-benzidine]. The reaction was stopped with acid and the absorbance was measured at 450 nm. The half-maximal inhibitory binding titer for each test sera was calculated.

The HIV-1 pseudovirus neutralization assay has been described elsewhere [54], [55]. This assay measures the reduction in luciferase expression following a single round of virus infection. Briefly, 3-fold serial dilutions of serum were performed in duplicate. Two hundred TCID50 of pseudovirus was added to each well and incubated for 1 h at 37 °C. TZM.bl cells were then added (1x10^4^/well) in 10% d-MEM medium. After 48 h (37 °C), 150ul of medium was added to 100ul of Bright-Glo luciferase reagent (Promega, Madison, WI), and luminescence was measured.

ADCC-mediated antibody responses were measured by ADCC GranToxiLux (GTL) [17] and tested against subtype AE HIV-1 recombinant A244_gD_negative_293F, 92TH023_293F_gD_negative, 1086.c_D7, B.MN_gp120_gD_negative, and Bal_gp120-coated cells. Participant sera were incubated with effector cells and gp120-coated target cells and ADCC was quantified as net percent granzyme B activity, which is the percent of target cells positive for GTL detected by flow cytometry. For each subject at each timepoint, percent granzyme B activity was measured at six dilution levels: 50, 250, 1250, 6250, 31,250 and 156,250 for each antigen. Peak activity<0% was set to 0%. A positive response was defined as peak activity greater than or equal to 8%.

The ADCC luciferase assay utilized a modified version of previously published procedure [56]. Briefly, CEM.NKRCCR5 cells were used as targets for ADCC luciferase assays after infection. Peripheral blood mononuclear cells (PBMCs) were used as effector cells at an effector-to-target ratio of 30:1. Target and effector cells were plated in opaque 96-well half-area plates and co-cultured with 5-fold serial dilutions of plasma. For each sample, percent specific killing was measured in two wells at dilutions 1:50, 1:200, 1:800, 1:3200, 1:12800, and 1:51200. Co-cultures were incubated for 6 h at 37°C in 5% CO_2_. The percentage of killing was calculated by determining the percent decrease of RLU in the test well. The RSV-specific monoclonal antibody Palivizumab and a cocktail of HIV-1 monoclonal Abs (A32, 2G12, CH44, and 7B2) were used as negative and positive controls, respectively. A positive response was defined as peak activity greater than or equal to 10% within the first two dilutions.

The RF-ADCC assay was also used to measure ADCC, and has been described elsewhere [57]. Briefly, EGFP-CEM-NKr-CCR5-SNAP target cells were stained with SNAP-Surface Alexa Fluor 647 with or without monomeric HIV-1 Bal (Clade B) gp120 (50 μg/ml). Gp120-sensitized EGFP-CEM-NKr-CCR5-SNAP target cells were serially diluted threefold starting at 1:100 through 1:1,968,300, together with control mAbs. A final volume of 100 μl/well of antibody/sera dilution (in triplicate) was added and incubated with sensitized targets for 15 min at room temperature. A total of 250,000 purified human effector PBMCs from healthy donor cells were added to each well at an effector/target ratio of 50:1. After 2 h of incubation, samples were analyzed on a BD LSRII Fortessa flow cytometer (BD Biosciences). Percentage cytotoxicity was defined as the percentage of EGFP-CEM-NKr-CCR5-SNAP target cells that lose GFP staining but retain CCR5-SNAP tag dye. Positivity was defined as percentage cytotoxicity greater than or equal to 22 percent. The results represent the average of samples tested in triplicate and normalized to the C11 positive control.

### 2.7 Statistical analysis

As this is a phase 1a first-in-human proof-of-concept study, no statistical estimation was done for sample size. The sample size was set to 60 (15 active vaccine recipients and 5 controls in each cohort) to allow adequate identification of potential toxicities and documentation of safety. Subjects who did not complete the study could be replaced at the discretion of the principal investigator. Besides the CD4i competition assay, all immunogenicity measurements were performed by the Comprehensive Antibody Vaccine Immune Monitoring Consortium (CAVIMC) at Duke University, and analysis of immunogenicity data and CD4 safety data was undertaken by the Vaccine Immunology Statistical Center (VISC). All statistical analysis was performed using the R programming language. Data manipulation and visualizations were generated using Tidyverse [58],linear mixed models were fit using the lme4 package [59],and comparisons using Barnard’s exact test (Z-pooled method) were performed using the Exact package [60].

## 3 Results

### 3.1 Study population

**Fig. 1:**
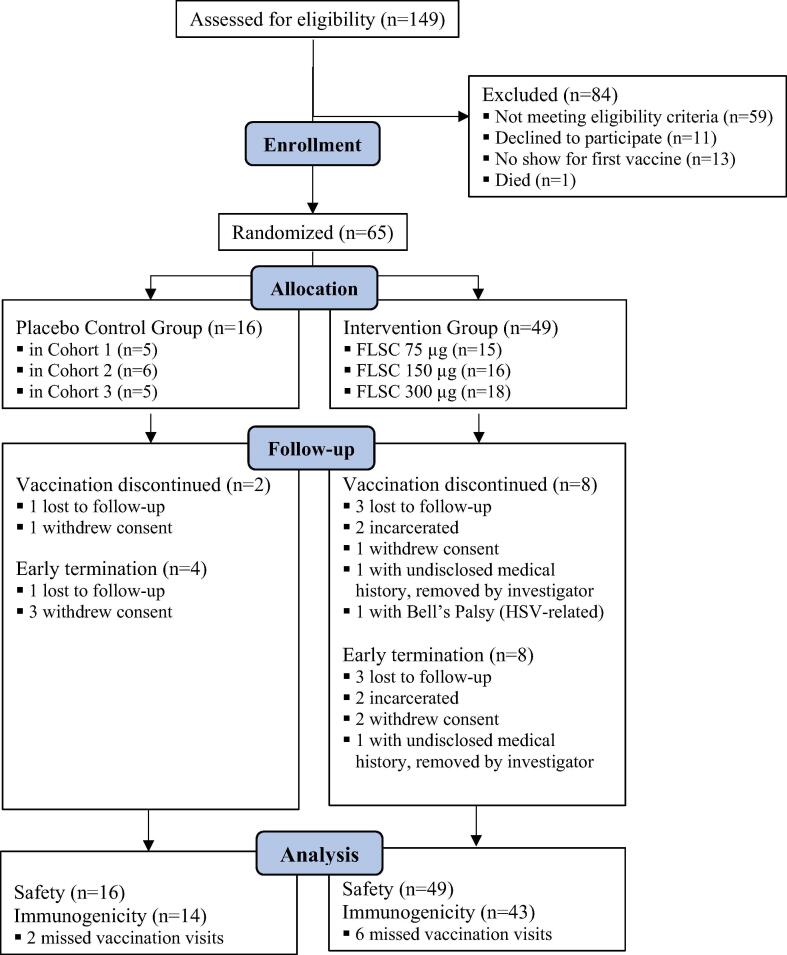
Study flow diagram.

Between October 19, 2015 and August 1, 2017, 149 volunteers were screened to achieve study enrollment numbers [Fig. 1]. Sixty-five participants were enrolled in the study. Forty-nine received vaccine and 16 received placebo. Overall, 241 intramuscular injections were administered. Fifty-three (82%) participants completed follow-up. Ten participants (8 vaccinees and 2 placebos) discontinued vaccination early [Fig. 1]. Twelve (8 vaccinees and 4 placebos) were terminated from the study early. Five participants who were lost to follow-up or removed were replaced. No subjects became infected with HIV during the study, and none seroconverted because of vaccination.

The median age of volunteers was 31 years [range 18–45] and 42% were women. The majority (66%) of volunteers were African American; 25% were Caucasian; and about 6% identified as Hispanic [Table 2]. Of the 65 participants enrolled, 57 received all four vaccinations as per protocol [Table 2]. IHV01 with corresponding dose assignment indicates vaccine recipient. For purpose of analysis, the control group combines placebo recipients from all three cohorts. Table 2 includes all participants who received at least one vaccination and shows the vaccination frequency by vaccination visit.

**Table 2:** Study population baseline characteristics.

			**T1**	**T2**	**T3**	**Total** **(n=65)**
		**Control** **(n=16)**	**75 µg** **(n=15)**	**150 µg** **(n=16)**	**300 µg** **(n=18)**	
Age (years)	Mean (SD)	29.8 (7.7)	33.8 (5.7)	31.3 (7.2)	30.6 (8.8)	31.3 (7.7)
	Median	30.5	36.0	31.5	31.0	31.0
	Range	18 - 45	22 - 41	21 - 43	19 - 43	18 - 45

Gender [n (%)]	Male	8 (50)	8 (53)	10 (62)	12 (67)	38 (58)
	Female	8 (50)	7 (47)	6 (38)	6 (33)	27 (42)

Race [n (%)]	Black or African American	11 (69)	11 (73)	8 (50)	13 (72)	43 (66)
	White or Caucasian	4 (25)	4 (27)	7 (44)	1 (6)	16 (25)
	Asian	0 (0)	0 (0)	1 (6)	2 (11)	3 (5)
	Others	1 (6)	0 (0)	0 (0)	2 (11)	3 (5)

Ethnicity [n (%)]	Hispanic or Latino(a)	2 (12)	1 (7)	0 (0)	1 (6)	4 (6)
	Not Hispanic or Latino(a)	14 (88)	14 (93)	16 (100)	17 (94)	61 (94)

Vaccine Frequency [n (%)]	Day 0	16 (100)	15 (100)	16 (100)	18 (100)	65 (100)
	Week 4	15 (94)	15 (100)	15 (94)	16 (89)	61 (94)
	Week 8	15 (94)	15 (100)	15 (94)	15 (83)	60 (92)
	Week 24	14 (88)	14 (93)	15 (94)	14 (78)	57 (88)

### 3.2 Reactogenicity

Eighty-one percent of vaccinations with IHV01 produced no localized or systemic reactions, which was no different from the placebo control group (80%). Table 3 summarizes the reactogenicity data of the study. Of those participants reporting local injection site pain and/or tenderness, most were mild with three moderate exceptions, all of which resolved and subsequent injections were tolerated without sequelae. No severe local reactions were reported for the placebo, 75 µg, or 150 µg dose groups**.**

**Table 3:** Summary of reactogenicity by injection number. All three vaccine dosing groups combined.

			**Events, No. (%)**															
			**First Dose**				**Second Dose**				**Third Dose**				**Fourth Dose**			
			**Vaccine**		**Control**		**Vaccine**		**Control**		**Vaccine**		**Control**		**Vaccine**		**Control**	
			**(n=49)**^1^		**(n=16)**^2^		**(n=46)**^3^		**(n=15)**^2^		**(n=45)**^4^		**(n=15)**^2^		**(n=43)**^5^		**(n=14)**^6^	
Local reactions																		
	Pain																	
		Any	6	(12.2)	1	(6.3)	3	(6.5)	2	(13.3)	7	(15.6)	3	(20.0)	4	(9.3)	1	(7.1)
		Grade 3	0		0		0		0		0		0		0		0	
	Erythema or induration																	
		Any	0		0		0		0		1	(2.2)	0		0		0	
		Grade 3	0		0		0		0		0		0		0		0	
	Tingling or numbness																	
		Any	0		0		0		0		1	0.022	0		0		0	
		Grade 3	0		0		0		0		0		0		0		0	
Systemic reaction																		
	Headaches		2	(4.1)	1	(6.3)	3	(6.5)	1	(6.7)	1	(2.2)	1	(6.7)	1	(2.3)	0	
	Pruritus		1	(2.0)	1	(6.3)	0		0		2	(4.4)	0		2	(4.7)	1	(7.1)
	Fever		2	(4.1)	1	(6.3)	0		0		0		0		0		0	
	Nausea		2	(4.1)	0		0		1	(6.7)	0		1	(6.7)	0		0	
	Fatigue		2	(4.1)	0		0		0		0		0		0		1	(7.1)
**Any systemic reaction**			**9**	**(18.4)**	**3**	**(18.8)**	**3**	**(6.5)**	**1**	**(6.7)**	**3**	**(6.7)**	**2**	**(13.3)**	**4**	**(9.3)**	**1**	**(7.1)**

1 One subject removed and replaced due to incarceration.

2 One subject lost to follow-up and replaced.

3 One subject removed and replaced due to undisclosed exclusion criteria, and one subject discontinued vaccination and replaced due to vaccine-unrelated Bell’s Palsy.

4 One subject withdrew consent and replaced.

5 Two subjects removed due to incarceration.

6 One subject withdrew consent.

Participants receiving the highest IHV01 dose tolerated majority of injections without any local injection site reactogenicity. Only four participants reported mild reactions after vaccination with the 300 µg dose over the course of the study, while one subject reported a mild reaction after the first immunization. One participant in this group had a severe local reaction (self-reported) after the first injection, which resolved by the time of the follow-up visit (14 days). Subsequent injections were tolerated without any sequelae.

### 3.3 Adverse events

The overall incidence of adverse events was not significantly different between the vaccine and control groups. Ninety-eight percent of vaccine-related adverse events (AEs) were either mild or moderate in severity. There were two grade 3 AEs possibly related to vaccine, which resolved without requiring medical care. One participant who received 300 µg vaccine experienced both grade 3 fever and grade 3 chills, after the 1st vaccination but tolerated subsequent vaccinations without recurrence of these symptoms. The most common adverse event (>10%) at least possibly related to vaccine by preferred term included injection site pain (31%), pruritus (10%), and headache (10%). The rates for the placebo group were 31% for injection site pain, 13% for pruritus, and 6% for headaches. There were no vaccine related SAEs, no intercurrent HIV infections (all subjects tested negative for HIV screening at the end of the study), no pregnancies, and no events that met stopping criteria. No participant had to stop immunizations due to a vaccine-related safety event. One participant who received 300 µg vaccine developed Bell’s palsy four weeks after his first vaccination and was discontinued from further immunization at the discretion of the investigator. The Bell’s palsy resolved spontaneously, was later found to be due to a herpes simplex virus infection and deemed not related to vaccination.

**Fig. 2:**
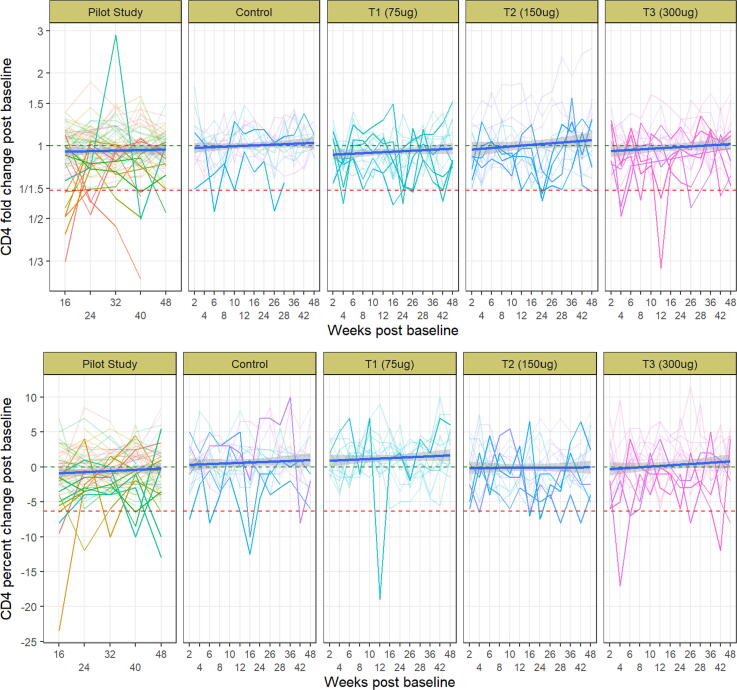
CD4 count fold change from baseline (in a log_10_ scale) and CD4 percentage difference frombaseline, by group. The red dashed lines represent 1.5-standard deviation declines for CD4 fold or percentage change. Each line is a single participant, and the solid blue line is the linear model fit, with grey shaded 95% confidence bands. Pilot study (healthy controls) results previously published (Stafford et al.) but included as a useful comparison for healthy controls in this study (labelled “Control”) and vaccinees. No decrease in CD4 counts or percentages noted in subjects. T1 = 75 µg group; T2 = 150 µg group; T3 = 300 µg group.

There were five total participants with either a simultaneous drop in both CD4 count and percentage outcomes or consecutive drops across two visits for one measure, but none of the vaccinees met the criteria established for unexplained CD4 loss (they either did not have drops in both measures of CD4 and CD4%, or drops were not sustained over four weeks). Of these five participants, two were in the placebo (control) group and the other three participants were equally distributed among the three treatment groups. To assess whether the vaccine generally induced declines in CD4 levels, we used linear, mixed-effects models (both unadjusted and adjusted for demographic variables) to compare 1) CD4 levels post-vaccination to pre-vaccination; and 2) changes in CD4 levels between the treatment groups and the pooled placebo group. No significant vaccine effects in CD4 count or CD4 percentage were found for either outcome (Fig. 2; Supplementary Table 2, Table 3).

### 3.4 Immunogenicity

**Fig. 3:**
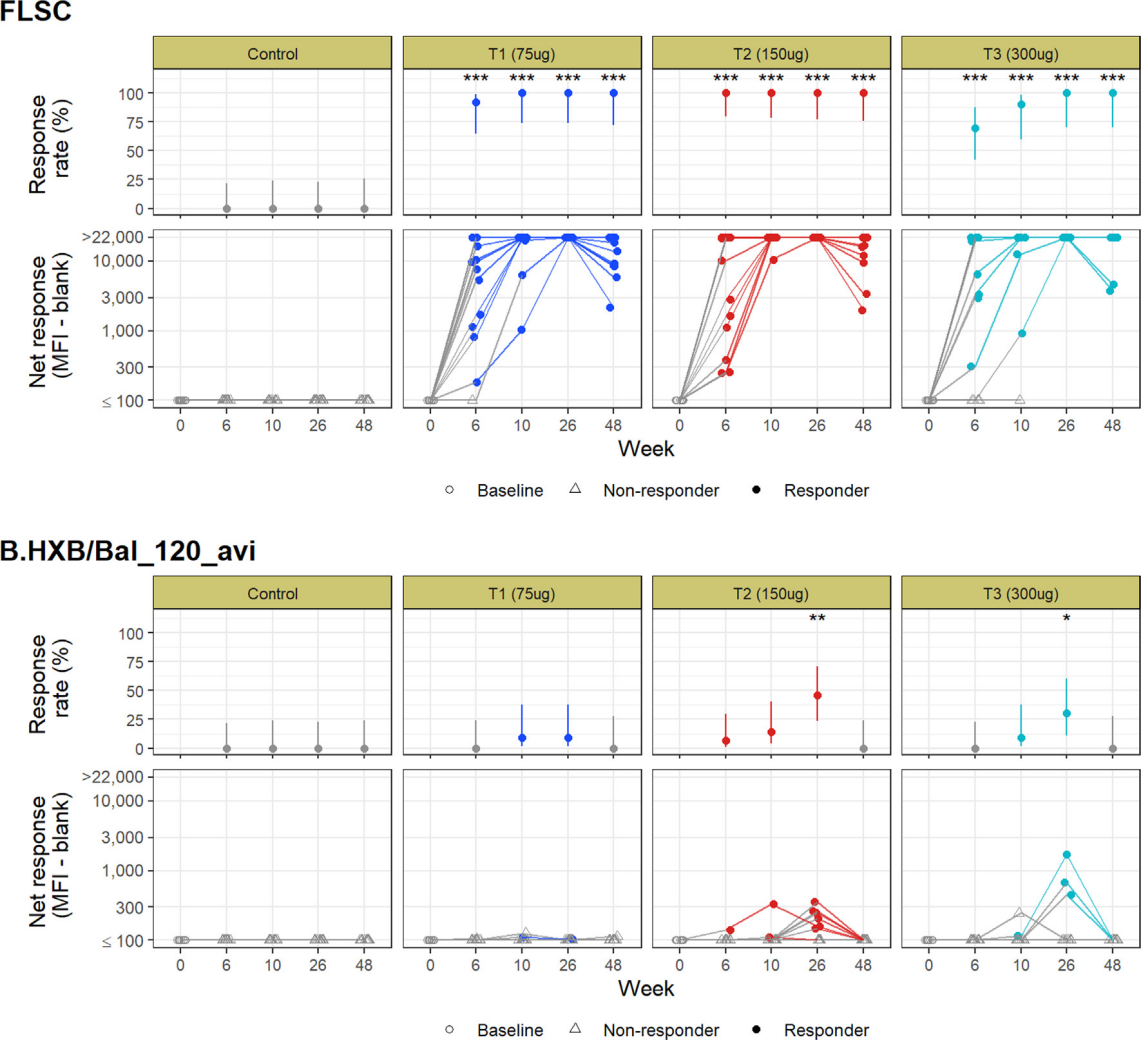
Binding antibody response rates and magnitudes (background-adjusted MFI minus blank) for each antigen and time point, by group. Response rates appear in the top panel, with accompanying Wilson score confidence intervals. Response rate testing significant comparing to control is noted (* p < 0.05, ** p < 0.01, *** p < 0.001). Net response magnitude displayed in the bottom panel, with open circles for baseline, open triangles for non-responders, and filled circles for responders, and lines connecting participants.

Responses to the FLSC component of IHV01 increased in all vaccination groups through the course of the immunization regimen [Fig. 3]. There were 100% vaccine response to FLSC in all three vaccine dose groups after the fourth vaccination (week 26); however, the 150 µg group achieved a 100% response rate after the second vaccination. There was a decrease in MFI binding titers in all three vaccine groups 24 weeks after the final vaccination (week 48) although response rates remained above 90%.

The majority of vaccinees developed serum antibody competition titers against the selected target CD4i epitopes. By week 26, 100% of the 150 µg and 300 µg groups developed antibodies that competed with either A32 or 17b for binding to the FLSC protein, compared with 64% in the 75 µg group (Table 4). By definition, these antibodies target Clusters A and C, respectively, of the CD4i epitope [61].

**Table 4:** Summary of anti-CD4i Epitope Response Rates.

**Week**	**Vaccine dose group**	**A32**		**17b**		**N12-i2**	
		**# Positive** **Total**	**% Positive**	**# Positive** **Total**	**% Positive**	**# Positive** **Total**	**% Positive**
Week 6	75 µg	12/15	80	1/15	7	3/15	20
	150 µg	7/15	47	8/15	53	4/15	27
	300 µg	9/15	60	8/15	53	9/15	60
Week 26	75 µg	9/14	64	7/14	50	5/14	36
	150 µg	13/14	93	14/14	100	13/14	93
	300 µg	12/12	100	10/12	83	6/12	50

**Fig. 4:**
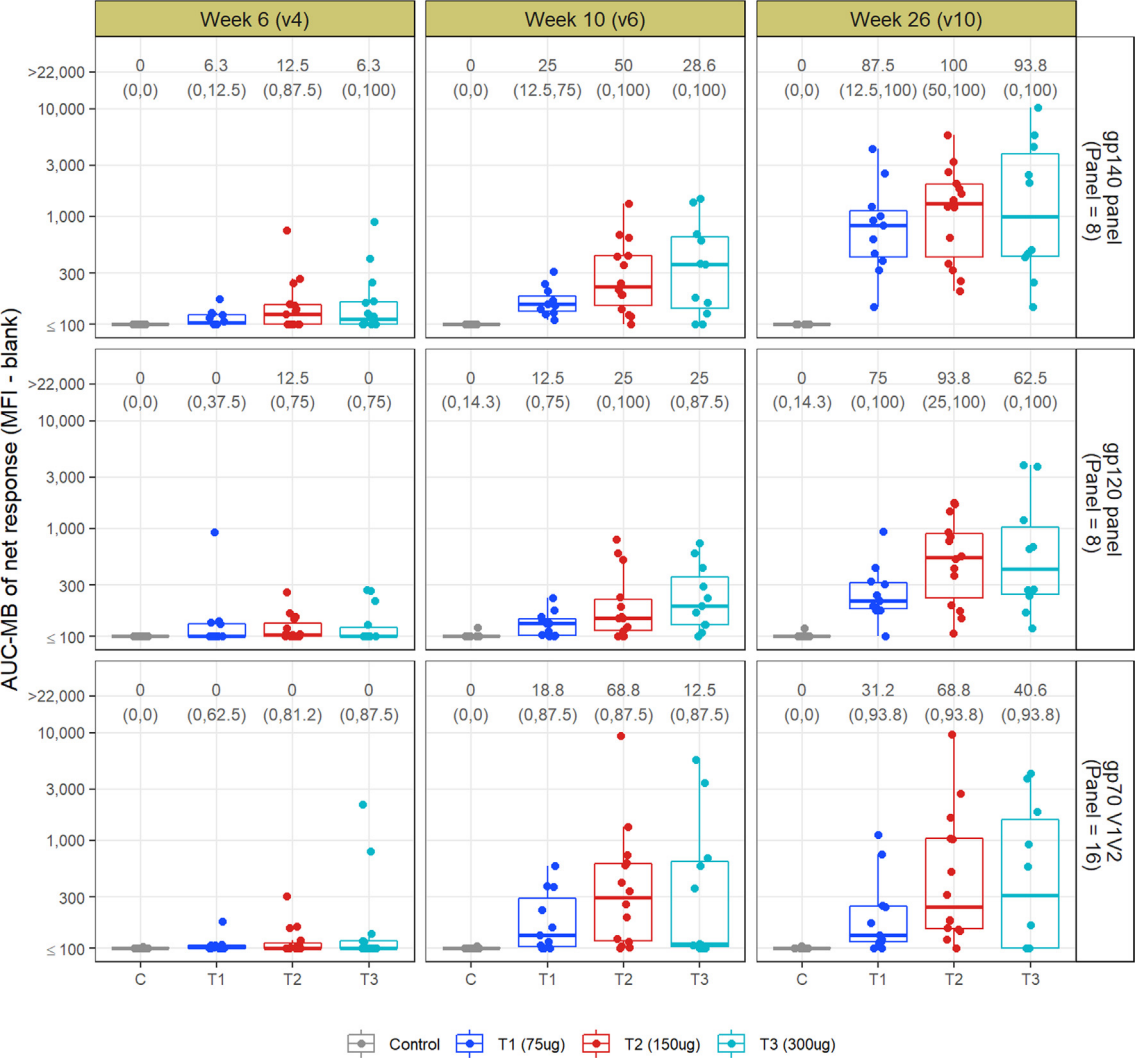
Magnitude-breadth AUC of the net binding antibody response against representative gp140, gp120 and V1V2 antigen breadth panels by group and time point. The box indicates the median and interquartile range (IQR); whiskers extend to the furthest point within 1.5 times the IQR from the upper or lower quartile. Values on the top represent the median and range of the response rate (%) within each antigen panel. C = placebo group; T1 = 75 µg group; T2 = 150 µg group; T3 = 300 µg group.

Humoral responses in all groups exhibited broad binding reactivity against CD4-nonliganded envelopes and gp70-scaffolded V1V2 loops [Fig. 4]. For all vaccine groups the AUC-MB scores increased during the vaccination regimen. After the final immunization (week 26), the highest response rates were observed in the 150 µg dose group, with 100%, 93.8% and 68.8% response rates for the gp140, gp120 and V1V2 panel, respectively. This group also exhibited the highest AUC-MB scores. Notably, 92% of the 150 µg group vaccinees were responders against the gp70 B.CaseA.V1V2 antigen (not shown). Responses to the latter antigen were predictive of reduced infection risk in the RV144 clinical trial [27].

**Fig. 5:**
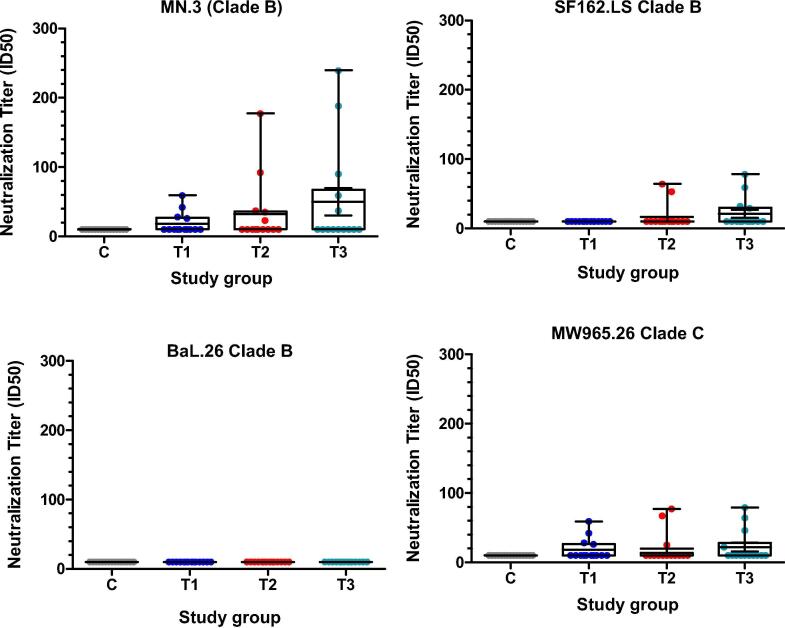
Neutralization titer ID50 against a panel of Clade B and C HIV pseudoviruses. The box indicates the median and interquartile range (IQR); whiskers extend to the furthest point within 1.5 times the IQR from the upper or lower quartile. Sera 2 weeks after last vaccination was tested for ability to neutralize MN.3, SF162.LS, BaL.26, and MW965.26; T1 = 75 µg group; T2 = 150 µg group; T3 = 300 µg group.

Neutralization titers were restricted to Tier 1 viruses and were low and variable across groups. At week 26, the highest titers were seen in the 300ug dose group [Fig. 5]. Surprisingly, there was no detectable neutralizing activity in any group against pseudoviruses expressing the Bal envelope, even though FLSC is based on BaL gp120 sequences.

ADCC activity was detected in all assay formats across the vaccination groups [Table 5]. In the RFADCC assay using HIV BaL gp120-coated target cells, response rates were 92.9–100% and peak median cytotoxicity was between 80 and 85% among groups. Similarly, in the GTL assay format using HIV BaL gp120-coated target cells there were 64.3, 85.7, 78.6 percent responders in the 75 µg, 150 µg and 300 µg dose groups, respectively at week 26. In this format there were also responders in all groups (week 26) against cells coated with HIV 1086c (Clade C) and HIV Mn (Clade B) gp120s (Table 5), but no responders against cells coated with A244 (Clade AE) or HIV 92TH023 (Clade E) envelopes (data not shown). In the luciferase assay format with infected cells, at week 26 responders were detected across groups in tests with HIV BaL, with 50% responders in the 150 µg dose group. Similar results were seen in assays with cells infected by HIV CM235 (Clade AE). Lower response rates were observed in assays with cells infected by HIV 1086 or HIV TV1 (Clade C).

**Table 5:** Summary of ADCC at week 26. Response rates and median peak % activity of positive responders are presented by treatment group.

			**C**	**T1**	**T2**	**T3**
**Assay**	**Target Variant**	**Measure**	**Result**			
RFADCC	BaL	% Responder	42.9	92.9**	100***	100***
		Peak % Activity	24	83.5	80.7	80.2
		AUTC	77.8	104	142.4	157.8
GTL	1086c_D7	% Responder	0	35.7*	50**	57.1***
		Peak % Activity	ND	12.4	14.8	16.7
		AUTC	ND	17.3	21.0	21.4
	B.MN_gDneg-gp120/293F	% Responder	0	14.3	14.3	35.7*
		Peak % Activity	ND	15.4	14.9	11.1
		AUTC	ND	22.5	22.3	20.2
	BaL	% Responder	0	64.3***	85.7***	78.6***
		Peak % Activity	ND	9.7	11.6	12.8
		AUTC	ND	9.5	16.4	17.1
	All variants (breadth)	AUTC (mean)	0.4	3.83	7.03	9.95
Luciferase	Ce1086_B2.LucR.T2A.ecto.293T	% Responder	0	ND	7.0	21.4
		Peak % Killing	ND	ND	11.49	12.48
		AUTC	ND	ND	11.03	3.89
	CM235-2.LucR.T2A/293T	% Responder	0	28.6*	50**	35.7*
		Peak % Killing	ND	21.4	19.3	37.7
		AUTC	ND	21.8	24.7	42.3
	TV1.21.LucR.T2A.ecto.293T*	% Responder	14.3	7.1	28.6	21.4
		Peak % Killing	14.3	12.6	15.3	20.8
		AUTC	5.8	14.7	12.5	13.1
	Bal.LucR.T2A.ecto/293T	% Responder	0	28.6*	50**	28.6*
		Peak % Killing	ND	15.9	19.6	19.0
		AUTC	ND	11.9	17.8	16.8
	All variants (breadth)	AUTC (mean)	1.6	3.45	6.23	1.84

Response rate were compared between vaccine groups and the control group using the using Barnard’s test (* p < 0.05, ** p < 0.01, *** p < 0.001).

## 4 Discussion

In this first-in-human phase 1a clinical trial, vaccination with IHV01 was well tolerated, safe, and immunogenic across all doses tested. Reactogenicity was similar between placebo and vaccine groups and decreased in both placebo and vaccine groups after the first vaccination. The most common side effect was pain at the injection site, followed by headache. The overall incidence of adverse events was not significantly different between the vaccine and the control groups, and there were no Grade 3 or 4 AEs that were definitely vaccine related. Overall, the safety and tolerability were similar to other HIV vaccines using HIV Env [62], [63], [64], [65], [66]**.**

As the vaccine product contains domains 1 and 2 of human CD4, special attention was paid the CD4 cell level (absolute values, cells/µl, and the percentage of CD4 cells) in this study. Prior to this study, we undertook a longitudinal study of CD4 cell counts over time in a healthy population, which served as a comparison [49]. In that study, we proposed that thresholds for declines using 1.5 SDs (50% in absolute count and 6.4% for CD4 percentage) allowed a small false-positive rate (~5%) that could maintain sensitivity for true adverse events in a clinical trial. This data was not available at the time of design of the current study, and in hindsight we included a more stringent safety monitoring for unexplained CD4 cell count decline (confirmed by assays at least 4 weeks apart) of greater than 30% and corroborated by similar CD4% decline (30%). However, no general trends in CD4 fluctuations were apparent in any group and no evidence of immune deficits were observed. These outcomes agreed with previous immunotoxicity studies carried out in cynomolgous macaques with IHV01 and the rhFLSC analogue, in which vaccinated animals showed no significant alterations in circulating CD4 + T cell levels [47] or T-cell functions.

The IHV01 vaccine was immunogenic, with all vaccinees developing responses to the FLSC protein by the end of the regimen. The responses included anti-gp120 antibodies that recognized envelope antigens and scaffolded V1V2 loops from diverse HIV strains [Table 4, Table 5 and Fig. 3, Fig. 4, Fig. 5]. Such broad reactivity was anticipated, as FLSC is designed to present highly conserved gp120 epitopes. Notably, as a matter of convention we used Env binding assays (performed by CAVIMC**;** see Methods) that are qualified to allow comparisons of clinical data from multiple HIV vaccine trials of Env-based immunogens. These assays utilize panels of Env antigens that are not in CD4-induced transition states and have variable and limited exposure of conserved CD4i epitopes. Consequently, these analyses may underestimate the breadth of Env cross-reactivity in IHV01 vaccine responses. Future testing with constrained Env protein panels will resolve this question.

Most HIV envelope-based vaccine candidates being developed to replicate viral structures have the potential to bind cell surface CD4 and/or CCR5 coreceptor in sequence. As an unformulated protein, FLSC will not bind CD4 but is able to bind CCR5 on cell surfaces in vitro [27]. In theory, such a property could influence the performance of IHV01. Nevertheless, the cross-competition ELISAs clearly demonstrated that the IHV01 vaccine responses in all groups recognized the highly conserved A32, 17b, and N12i2 CD4i epitopes (Table 4) in the coreceptor binding site. Further, there were no safety signals indicative of CCR5-related perturbations. These data argue against extensive CCR5 occupation by the formulated FLSC, although the possibility that a minor amount occurred post-vaccination cannot be eliminated.

Of note, the neutralizing responses raised in the vaccinees exhibited potency and breadth patterns that were lower and narrower than what was detected in nonhuman primates given IHV01 or rhFLSC variants [47], [30], [31]. Even though IHV01 included the gp120 from the BaL strain, no neutralizing activity was detected against the HIV BaL-pseudotyped viruses under the assay conditions employed (Figure 5). Whether different adjuvant formulations, vaccine doses or immunization schedules will improve neutralizing titers may be considered for future studies. The findings from this study indicate that immune responses against type-specific neutralizing epitopes (e.g. those on BaL gp120) were dampened while those against conserved, non-neutralizing epitopes that guide other humoral effector functions were favored.

Consistent with the above interpretation, the vaccine responses in all groups exhibited cross-reactive ADCC activity, extending to HIV BaL, in multiple assay formats [Table 5]. This outcome follows our previous studies of rhFLSC vaccination in rhesus macaques [30], [31], where ADCC correlated with reduced risk of infection. The functional data are also consistent with the competition ELISA data reflecting the presence of plasma antibodies to Cluster A and coreceptor binding site epitopes. Antibodies with such specificities exhibit potent ADCC activity in the same assay formats [10], [13], [17], [57]. As noted in the above sections, the only reported impact of cross-reactive non-neutralizing responses with Fc-mediated effector functions on HIV infection risk has been to reduce probability of acquisition or virus spread. Thus, the IHV01 responses are potentially advantageous for HIV prevention strategies.

## 5 Conclusion

In this first-in-human phase I clinical trial, we found that vaccination with IHV01 was safe and well-tolerated. There were no Grade 3 or 4 AEs definitely related to vaccine. In addition, there were no decreases in CD4 count or CD4 percentage after vaccination. There was no HIV seroconversion during or because of vaccination. The IHV01 vaccine was immunogenic in accordance with its design, raising antibodies against FLSC and highly conserved CD4i epitopes. The elicited antibodies were broadly cross-reactive with gp120, gp140, and V1V2 domains representing multiple clades of HIV-1. Finally, the vaccine responses mediated cross-reactive humoral effector functions against HIV in vitro. IHV01 may be considered as a component of future HIV vaccination strategies.

## CRediT authorship contribution statement

Joel V. Chua and Mohammad M. Sajadi drafted the manuscript and supervised its completion. Joel V. Chua, Bruce L. Gilliam, and Charles Davis are the principal clinical investigators that conducted the study. Jennifer Husson, Amy Nelson, Ka Wing J. Lam, Lydiah Mutumbi cared for the participants and collected the data. Bryan T Mayer, Dan Hong, William Fulp, Celia Mahoney, Monica Gerber, and Raphael Gottardo conducted the statistical analysis. Ilia Prado, Robin Flinko, Kelli Greene, Hongmei Gao, Nicole Yates, Guido Ferrari, Georgia Tomaras, and David Montefiori conducted the immunogenicity work. Bruce L. Gilliam, Timothy Fouts, Jennifer A. Swartz, Anthony L. DeVico, George K Lewis, and Robert C Gallo conceptualized and designed the study.

## Declaration of Competing Interest

The authors declare that they have no known competing financial interests or personal relationships that could have appeared to influence the work reported in this paper.
