# Supplementary material for: Safety and immunogenicity of an HIV-1 gp120-CD4 chimeric subunit vaccine in a phase 1a randomized controlled trial
Source: Vaccine. 2021 Jun 29;39(29):3879–91. doi: 10.1016/j.vaccine.2021.05.090 (PMC8224181; doi:10.1016/j.vaccine.2021.05.090)
Supplement: Supplementary data 1 [file mmc1.docx]

**Supplemental Table 1:** AIDS Reagent Program panel of gp120, gp140, and V1V2 antigens.

|  |  |  |
| --- | --- | --- |
|  |  |  |
| **Antigen** | **Protein** | **Clade** |
| 51802_D11gp120 | gp120 ARP | A |
| B.6240_D11gp120 | gp120 ARP | B |
| BORI_D11gp120 | gp120 ARP | B |
| TT31P.2792_D11gp120 | gp120 ARP | B |
| 254008_D11gp120 | gp120 ARP | CRF01_AE |
| A244 D11gp120 | gp120 ARP | CRF01_AE |
| BJOX002_D11gp120 | gp120 ARP | CRF07_BC |
| CNE20_D11gp120 | gp120 ARP | CRF07_BC |
| 9004S.gp140C | gp140 ARP | A |
| RHPA4259_C7.gp140C | gp140 ARP | B |
| SC42261_gp140 | gp140 ARP | B |
| WITO4160.gp140C | gp140 ARP | B |
| BF1266_gp140C | gp140 ARP | C |
| C.CH505TF_gp140 | gp140 ARP | C |
| 1086C gp140C | gp140 ARP | C |
| AE.01.con_env03 | gp140 ARP | CRF01_AE |
| gp70-191084_B7 V1V2 | V1V2 ARP | A |
| gp70-62357.14 V1V2 | V1V2 ARP | B |
| gp70-700010058 V1V2 | V1V2 ARP | B |
| gp70-RHPA4259.7 V1V2 | V1V2 ARP | B |
| gp70-TT31P.2F10.2792 V1V2 | V1V2 ARP | B |
| gp70-001428.2.42 V1V2 | V1V2 ARP | C |
| gp70-7060101641 V1V2 | V1V2 ARP | C |
| gp70-96ZM651.02 V1V2 | V1V2 ARP | C |
| gp70-BF1266_431a_V1V2 | V1V2 ARP | C |
| gp70-CAP210.2.00.E8 V1V2 | V1V2 ARP | C |
| gp70-Ce1086_B2 V1V2 | V1V2 ARP | C |
| gp70-C2101.c01_V1V2 | V1V2 ARP | CRF01_AE |
| gp70-CM244.ec1 V1V2 | V1V2 ARP | CRF01_AE |
| gp70-BJOX002000.03.2 V1V2 | V1V2 ARP | CRF07_BC |
| gp70_B.CaseA_V1_V2 | V1V2 ARP | B |
| gp70-TV1.21 V1V2 | V1V2 ARP | C |

**Supplemental Table 2:** CD4 Count Model.

| **Variable** | **Level** | **Est (95% CI)** | **P value** | **Overall P value** | **Est (95% CI)** | **P value** | **Overall P value** |
| --- | --- | --- | --- | --- | --- | --- | --- |
| Group | C | Reference | --- | 0.8861 | Reference | --- | 0.8084 |
| Group | T1 | -0.014 (-0.110, 0.081) | 0.7693 |  | -0.033 (-0.122, 0.056) | 0.4661 |  |
| Group | T2 | 0.010 (-0.085, 0.104) | 0.8437 |  | 0.010 (-0.077, 0.096) | 0.8279 |  |
| Group | T3 | -0.025 (-0.117, 0.066) | 0.5898 |  | -0.003 (-0.088, 0.082) | 0.9493 |  |
| Pre-post | Pre-Vaccination | Reference | --- |  | Reference | --- |  |
| Pre-post | Post-Vaccination | 0.000 (-0.029, 0.029) | 0.9986 |  | 0.000 (-0.029, 0.028) | 0.9847 |  |
| Group:pre-post | C: Pre-Vaccination | Reference | --- | 0.6428 | Reference | --- | 0.6487 |
| Group:pre-post | T1: Post-Vaccination | -0.022 (-0.064, 0.019) | 0.2863 |  | -0.022 (-0.063, 0.019) | 0.2911 |  |
| Group:pre-post | T2: Post-Vaccination | 0.001 (-0.040, 0.042) | 0.9539 |  | 0.002 (-0.039, 0.042) | 0.9376 |  |
| Group:pre-post | T3: Post-Vaccination | -0.012 (-0.052, 0.028) | 0.5638 |  | -0.011 (-0.051, 0.029) | 0.5995 |  |
| Age |  |  |  |  | 0.006 (0.002, 0.010) | 0.0088 |  |
| Gender | Male |  |  |  | Reference | --- |  |
| Gender | Female |  |  |  | 0.103 (0.043, 0.163) | 0.0013 |  |
| Race | Caucasian |  |  |  | Reference | --- | 0.6879 |
| Race | African American |  |  |  | -0.016 (-0.084, 0.051) | 0.6366 |  |
| Race | Other |  |  |  | -0.054 (-0.178, 0.070) | 0.3957 |  |

Legend: C = Control; T1 = 75 µg vaccine; T2 = 150 µg vaccine; T3 = 300 µg vaccine.

**Supplemental Table 3:** CD4 Percent Model.

| **Variable** | **Level** | **Est (95% CI)** | **P value** | **Overall P value** | **Est (95% CI)** | **P value** | **Overall P value** |
| --- | --- | --- | --- | --- | --- | --- | --- |
| Group | C | Reference | --- | 0.8843 | Reference | --- | 0.9542 |
| Group | T1 | 1.729 (-3.912, 7.371) | 0.55 |  | 0.044 (-5.090, 5.179) | 0.9865 |  |
| Group | T2 | 1.375 (-4.175, 6.925) | 0.6288 |  | 0.876 (-4.137, 5.889) | 0.733 |  |
| Group | T3 | -0.076 (-5.470, 5.317) | 0.9779 |  | 1.189 (-3.739, 6.117) | 0.6378 |  |
| Pre-post | Pre-Vaccination | Reference | --- |  | Reference | --- |  |
| Pre-post | Post-Vaccination | 0.649 (-0.454, 1.752) | 0.2494 |  | 0.640 (-0.463, 1.743) | 0.2555 |  |
| Group:pre-post | C:Pre-Vaccination | Reference | --- | 0.381 | Reference | --- | 0.3888 |
| Group:pre-post | T1:Post-Vaccination | 0.604 (-0.974, 2.183) | 0.4534 |  | 0.614 (-0.965, 2.192) | 0.4462 |  |
| Group:pre-post | T2:Post-Vaccination | -0.727 (-2.295, 0.840) | 0.3634 |  | -0.715 (-2.282, 0.853) | 0.372 |  |
| Group:pre-post | T3:Post-Vaccination | -0.425 (-1.961, 1.111) | 0.5879 |  | -0.398 (-1.934, 1.138) | 0.6117 |  |
| Age |  |  |  |  | 0.409 (0.159, 0.659) | 0.0022 |  |
| Gender | Male |  |  |  | Reference | --- |  |
| Gender | Female |  |  |  | 3.309 (-0.320, 6.937) | 0.0792 |  |
| Race | Caucasian |  |  |  | Reference | --- | 0.1793 |
| Race | African American |  |  |  | -1.160 (-5.279, 2.959) | 0.5831 |  |
| Race | Other |  |  |  | -7.056 (-14.583, 0.471) | 0.0714 |  |

Legend: C – Control; T1 = 75 µg vaccine; T2 = 150 µg vaccine; T3 = 300 µg vaccine.
